# *C*-Glycoside-Metabolizing Human Gut Bacterium, *Dorea* sp. MRG-IFC3

**DOI:** 10.4014/jmb.2308.08021

**Published:** 2023-09-21

**Authors:** Huynh Thi Ngoc Mi, Santipap Chaiyasarn, Heji Kim, Jaehong Han

**Affiliations:** Metalloenzyme Research Group and Department of Plant Science and Technology, Chung-Ang University, Anseong 17546, Republic of Korea

**Keywords:** C-C bond cleavage, *C*-glycoside, *Dorea* sp. MRG-IFC3, gut metabolism, puerarin

## Abstract

Biochemical gut metabolism of dietary bioactive compounds is of great significance in elucidating health-related issues at the molecular level. In this study, a human gut bacterium cleaving C-C glycosidic bond was screened from puerarin conversion to daidzein, and a new, gram-positive *C*-glycoside-deglycosylating strain, *Dorea* sp. MRG-IFC3, was isolated from human fecal sample under anaerobic conditions. Though MRG-IFC3 biotransformed isoflavone *C*-glycoside, it could not metabolize other *C*-glycosides, such as vitexin, bergenin, and aloin. As evident from the production of the corresponding aglycons from various 7-*O*-glucosides, MRG-IFC3 strain also showed 7-*O*-glycoside cleavage activity; however, flavone 3-*O*-glucoside icariside II was not metabolized. In addition, for mechanism study, *C*-glycosyl bond cleavage of puerarin by MRG-IFC3 strain was performed in D_2_O GAM medium. The complete deuterium enrichment on C-8 position of daidzein was confirmed by ^1^H NMR spectroscopy, and the result clearly proved for the first time that daidzein is produced from puerarin. Two possible reaction intermediates, the quinoids and 8-*d*ehydrodaidzein anion, were proposed for the production of daidzein-8*d*. These results will provide the basis for the mechanism study of stable C-glycosidic bond cleavage at the molecular level.

## Introduction

Microbial biotransformation plays a key role in the ecosystem and environmental sustainability. The conversion of organic and inorganic materials accompanies the transfer of solar energy to bioenergy while also providing organisms with chemical tools for responding to environmental changes. Particularly, the metabolism of natural products in the human gut offers new insights regarding human health and new chemical conversion processes [1, 2]. The human gut is now seen as a new microbial environment where a wide range of symbiotic interactions occur between human cells and gut microbiota. Similar to the ways in which environmental microorganisms respond to environmental changes, human gut bacteria also adjust their biochemical metabolisms according to changes in the gut environment resulting from disease, physiology, diet, and other stimuli.

A *C*-glycoside is a compound with a carbohydrate unit attached to an aglycone or another carbohydrate unit through a glycosidic C-C bond. Bioactive *C*-glycosides are important natural products related to human health, as exemplified by their use in antibiotics, as well as their neuroprotective, anti-diabetic, and anti-Alzheimer activities [3-6]. While *C*-glycosides have great potential for wider biotechnological application, further studies on the metabolism and absorption of *C*-glycosides in the human gut are necessary to link their clinical efficacy and in vitro biological activity [7]. For example, although barbaloin is not purgative, its gut metabolite, aloe-emodin, is a purgative agent [8].

So far, no evidence of C-glycosidic bond cleavage by phase I and II metabolisms in the liver has been found. However, many gut and soil bacteria are known to cleave the C-C bond to metabolize *C*-glycosides [9, 10], including *Lactococcus* sp. MRG-IFC-1, *Enterococcus* sp. MRG-IFC-2 [11], *Lachnospiraceae* strain CG19-1 [12], *Enterococcus faecalis* W12-1 [13], strain PUE [14], *Enterococcus* sp. 45 [15], *Eubacterium* sp. strain BAR [8], *Bacteroides* sp. MANG [16], and *Microbacterium* sp. 5-2b [10]. These bacteria are known to metabolize various *C*-glycosides, but an indisputable biochemical reaction mechanism of the glycosidic C-C bond cleavage has not yet been established. Up to now, the genes responsible for the glycosidic C-C bond cleavage have only been reported from strains CG19-1 and PUE.

On the other hand, significant progress has recently been reported regarding *C*-glycoside metabolism. Biological *C*-glycoside metabolism begins with the oxidation of glycosyl moiety, regardless of substrate diversity, resulting in the 3-oxo-*C*-glycoside, which is further metabolized by other enzymes responsible for the glycosidic C-C bond cleavage [10, 17]. The first step of glucose oxidation is achieved by enzymes and requires a cofactor, such as NAD^+^ or FAD. However, the enzyme reaction mechanism of the next reaction step of the C-C bond cleavage is still rather unclear [18, 19].

In this work, we report the characteristics and reactivity of a new anaerobic bacterium that metabolizes puerarin to daidzein to investigate the reaction mechanism of the glycosidic C-C bond cleavage at the molecular level. In addition, puerarin biotransformation in deuterium oxide medium was also performed to identify the reaction intermediate.

## Materials and Methods

### Chemicals

Puerarin (daidzein-8-*C*-glucoside), daidzin (daidzein-7-*O*-glucoside), icariin, and bergenin were from Sejin CI (Korea). D_2_O was obtained from Merck. Apigetrin (apigenin-7-*O*-glucoside), glycitin (glycitein 7-*O*-glucoside), genistin (genistein 7-*O*-glucoside), ononin (formononetin 7-*O*-glucoside), and sissotrin (biochanin A 7-*O*-glucoside) were purchased from Indofine Chemical Co. (USA). Aloin was obtained from Alfa Aesar (Thermo Fisher Scientific, USA), and vitexin (apigenin-8-*C*-glucoside) was obtained from Fluka (Sigma-Aldrich Co., USA). HPLC-grade methanol was purchased from Burdick & Jackson (USA), and ethyl acetate and acetic acid were from Fisher (USA). Gifu anaerobic medium (GAM) from Nissui Pharmaceutical Co. (Japan) was used for isolation and growth media. GAM broth was prepared according to the manufacturer’s instructions, and the GAM plate contained 15 g/l agar in GAM broth. All other chemicals were of analytical reagent grade.

### Bacterial Culture and 16S rDNA Sequencing

The experimental protocol was evaluated and approved by the Institutional Review Board of Chung-Ang University (Approval No. 1041078-201502-BR-029-01). All the experimental procedures, including screening, isolation, and identification of bacteria, were performed under anaerobic conditions (CO_2_ 5%, H_2_ 10%, N_2_ 85%) at 37°C, except for the metabolite analysis after biotransformation [11]. Analysis of the 16S rRNA gene sequence was performed to identify bacterial strains via the sequencing service at Macrogen (Korea). The 16S rRNA gene was amplified by PCR with universal bacteria primers 27 F (5'-AGAGTTTGATCMTGGCTCAG-3') and 1492R (5'-TACGGYTACCTTGTTACGACTT-3'), and analyzed using NCBI’s BLASTN tool to identify the bacterial strains from sequence similarity [20]. Phylogenetic trees were constructed by the neighbor-joining method using the MEGA X program [21]. The isolate was deposited with the Korean Collection for Type Cultures (Korea) under the accession number KCTC 25707.

### Growth Curve and pH Change

A single colony was picked from the GAM plate subculture and inoculated into 3 ml of GAM broth overnight. Then, 200 μl of the prepared culture was transferred to 20 ml of GAM broth. The bacteria culture was incubated under anaerobic conditions at 37°C. The optical density at 600 nm was recorded by a UV-Vis spectrophotometer S-3100 (Scinco, Korea) and pH changes were measured by using a pH meter (Ohaus pH meter, ST2100, Switzerland). All measurements were performed in triplicate.

### Biotransformation Study

Generally, 20 μl of each substrate (10 mM in DMF) was added to 1 ml bacteria culture, and reached OD_600nm_ 0.6 ~ 0.9 under anaerobic conditions at 37°C. Aliquots of the reaction (100 μl) were taken and allocated into Eppendorf tubes following the scheduled reaction times (0, 24, 48, 72, and 96 h, respectively). The allocated culture was extracted with 1 ml ethyl acetate, and 800 μl supernatant was collected after vortexing and centrifuging at 10,770 ×*g* for 10 min. The organic phase was then dried under vacuum, and the dried residue was dissolved in 100 μl MeOH filtered through a 0.2 μl PTFE (Polytetrafluoroethylene) filter (Advantec, Japan) for the chromatography analysis by TLC and HPLC.

### Time-Dependent Biotransformation of Puerarin

Puerarin and daidzin were used as substrates for time-dependent biotransformation under anaerobic conditions. Bacteria were grown to OD at 600 nm in 15 ml of GAM media, and 100 μl of each substrate (10 mM in DMF) was added to a 5 ml bacteria culture. Thereafter, 100 μl of the reaction mixture was taken and allocated into Eppendorf tubes every 15 min. The allocated media were extracted with 1 ml ethyl acetate, and 800 μl supernatant was collected after vortexing and centrifuging at 10,770 ×*g* for 10 min. The organic phase was then dried under vacuum, and the dried residue was dissolved in 100 μl MeOH filtered through a 0.2 μl PTFE filter and analyzed by HPLC. The experiment was carried out in triplicate.

### Puerarin Biotransformation in D_2_O Medium

Two media were prepared by dissolving 2.95 g of GAM in 50 ml of D_2_O (99% D; Sigma-Aldrich), or distilled water. Both were filtered through a 0.2 μl cellulose acetate syringe filter, and bacterial culture (500 μl) and 11 mg (0.053 mmol) puerarin were added and incubated for 24 h. Thereafter, the reaction mixture was extracted thrice with 50 mL of ethyl acetate (washed with H_2_O). The extract was evaporated to dryness by vacuum, dissolved in DMSO, and ^1^H-NMR was taken on a 600 MHz Varian NMR system. For comparison of the reaction times, the aliquots from each medium were taken at 0, 1.5, and 3 h, respectively. The analytes were prepared as described above for HPLC analysis.

### HPLC Analysis

HPLC analysis was conducted by UHPLC-DAD, a Dionex Ultimate 3000 UHPLC system (Thermo Fisher Scientific), with a kinetex C18 column (1.7 μm particle size; 100 × 2.1 mm i.d., Phenomenex, USA) at a temperature of 35°C. The flow rate was performed at 0.2 ml with the mobile phase consisting of 0.1% acetic acid (v/v) in water (A) and acetonitrile (B). The elution profile started with solvent B from 5 to 55% in 20 min, followed by holding at 55% for 5 min, with linear increase from 55 to 100% in 5 min, and then holding for 3 min. The injection volume was 1.0 μl and the wavelength was recorded: puerarin, and daidzin at 250 nm; begernin, icariin, and icariside II at 270 nm; genistin and ononin at 256 nm; vitexin at 290 nm, and aloin at 300 nm with UV spectrum monitoring in the range of 200-400 nm. Program setup, data collection and analysis were performed by using Chromelon Chromatography Data System software version 6.8 (Thermo Fisher Scientific).

### HPLC-MS Analysis

Chromatography was carried out on Hypersil gold 3 μm (100 × 2.1 mm) with the temperature at 25°C. The flow rate was performed at 0.2 ml with the mobile phase consisting of 0.1% formic acid (v/v) in water (A) and 0.1%formic acid in acetonitrile (C). The elution profile started with solvent C from 5% for 5 min, followed by linear increase to 20% in 5 min, then from 20 to 80% in 2 min, followed by holding at 80% for 3 min, and then decrease to 5% in 2 min. The column effluent was introduced into the mass spectrometer using electrospray ionization in negative mode.

## Results

### A New Puerarin-Metabolizing Bacterium

**Fig. 1:**
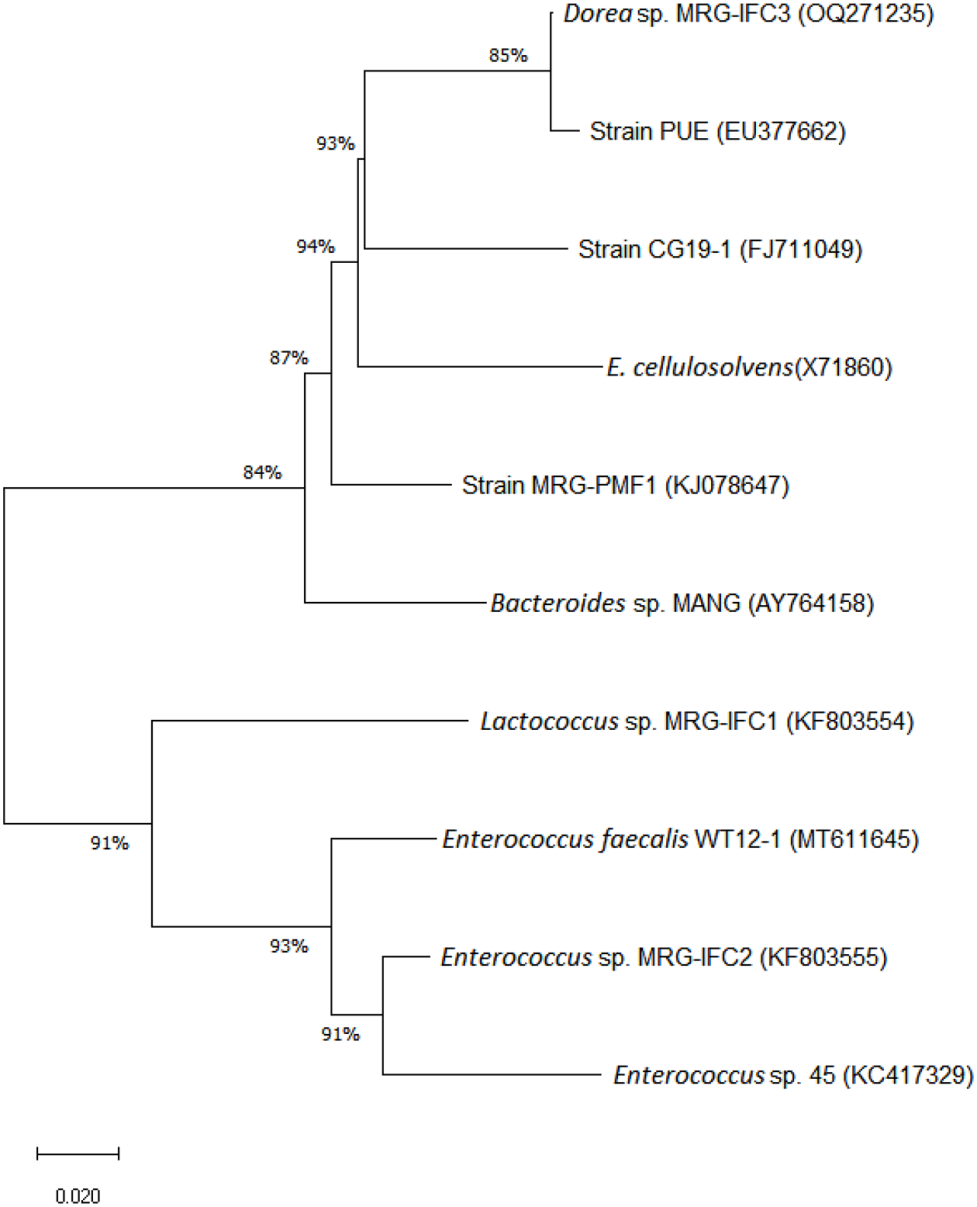
Phylogenetic tree showing the relationship between *Dorea* sp. MRG-IFC3. *Lactococcus* sp. MRG-IFC-1 (KF803554), *Enterococcus* sp. MRG-IFC-2 (KF803555), *Lachnospiraceae* strain CG19-1 (FJ711049), *Enterococcus faecalis* W12-1 (MT611645), strain PUE (EU377662), *Enterococcus* sp. 45 (KC417329), *Bacteroides* sp. MANG (AY764158) and *Eubacterium cellulosolvens* (X71860). The outgroup for the phylogenetic analysis was *Blautia* sp. MRG-PMF1 (KJ078647).

The puerarin-metabolizing gut bacterium was isolated from the human fecal sample under anaerobic conditions. The bacterium was named as strain MRG-IFC3 (KCTC 25707) and was a strictly anaerobic, short, rod-shaped gram-positive bacterium (Fig. S1, Table S1). The 16S rRNA gene of the isolate was sequenced for bacterial identification and the partial DNA sequence was deposited in GenBank (Accession No. OQ271235). From Database Resources of the National Center for Biotechnology Information, the partial 16S rRNA gene (1465 bp) of MRG-IFC3 strain showed 99.93% similarity to the gram-positive *Dorea longicatena* strain VE303-06 (Accession No. CP094679) by Nucleotide Blast [22]. Therefore, the isolated bacterium was designated as *Dorea* sp. MRG-IFC3 strain. The 16S rRNA gene sequence of *Dorea* sp. MRG-IFC3 was compared with those of other human intestinal bacteria known to metabolize *C*-glycosides (Fig. 1). According to phylogenetic analysis, *Lactococcus* sp. and *Enterococcus* spp. formed a separate group from the other bacteria. *Dorea* sp. MRG-IFC3 appeared to be closely related to the strain PUE, and strain CG19-1 was also close to the two strains MRG-IFC3 and PUE. Considering the position of the outstanding group, the aryl methyl ether-metabolizing gut bacterium MRG-PMF1 [23], the phylogenetic relationship between *Bacteroides* sp. MANG and other *C*-glycoside-metabolizing bacteria seemed very scant.

**Fig. 2:**
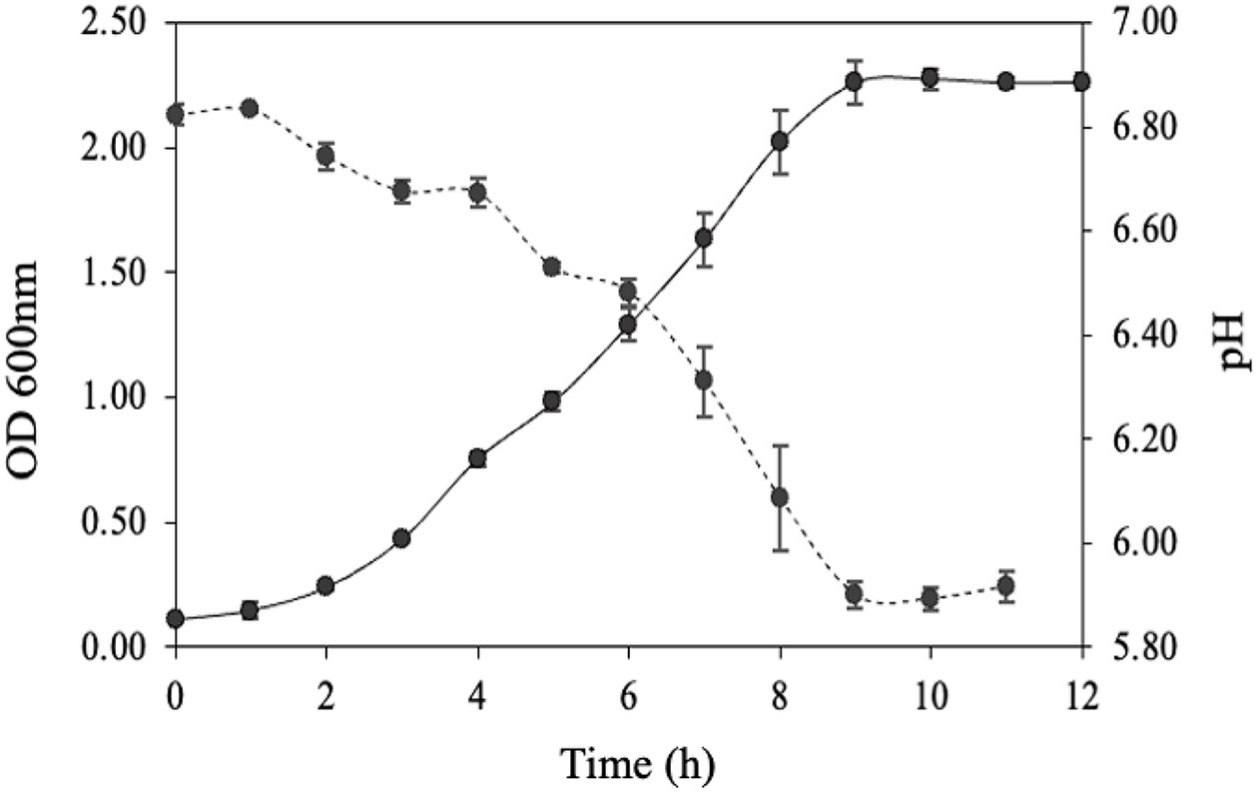
Growth curve and pH changes of *Dorea* sp. MRG-IFC3. Cell growth was measured by monitoring OD_600_ in GAM medium at 37°C under anaerobic conditions.

The growth curve of MRG-IFC3 strain was monitored in GAM medium at 37°C under anaerobic conditions. MRG-IFC3 stayed at lag phase for the first 3 h and reached stationary phase in 9 h of incubation time. During the growth of MRG-IFC3 strain, the pH value decreased from 6.8 to 5.9 (Fig. 2).

### Reactivity and Biotransformation Kinetics of *Dorea* sp. MRG-IFC3

The reactivity of strain MRG-IFC3 was investigated with various C- and *O*-glycosides, because it was still not clear whether the enzyme responsible for metabolizing *C*-glycosides could metabolize *O*-glycosides (Table 1, Figs. S2, S3, and S4). Apigetrin and ononin were completely metabolized to apigenin and formononetin, respectively, in 24 h. Sissotrin was converted to biochanin A in 3 h, faster than other *O*-glycosides. Icariin was completely metabolized to icariside II in 96 h; however, icariside II was inert to biotransformation, and further conversion to icaritin was not observed. In the previous study [24], icariin was metabolized to icariside II by MRG-ICA-B, MRG-ICA-E, and MRG-PMF1, and icariside II was then converted to icaritin and desmethylicaritin by MRG-PMF1. In summary, *Dorea* sp. MRG-IFC3 could metabolize most flavonoid *O*-glycosides, whereas only puerarin, an isoflavone-*C*-glycoside, was converted to aglycone.

*Dorea* sp. MRG-IFC3 could not convert aloin, vitexin, or bergenin, and thus we found that the strain showed high substrate specificity for *C*-glycoside metabolism, similar to *Lactococcus* sp. MRG-IFC1. Meanwhile, *Lachnospiraceae* strain CG19-1 was reported to exhibit a broad substrate spectrum of *C*-glycoside biotransformation by converting isoflavone, flavone, and xanthone *C*-glycosides [12].

**Fig. 3:**
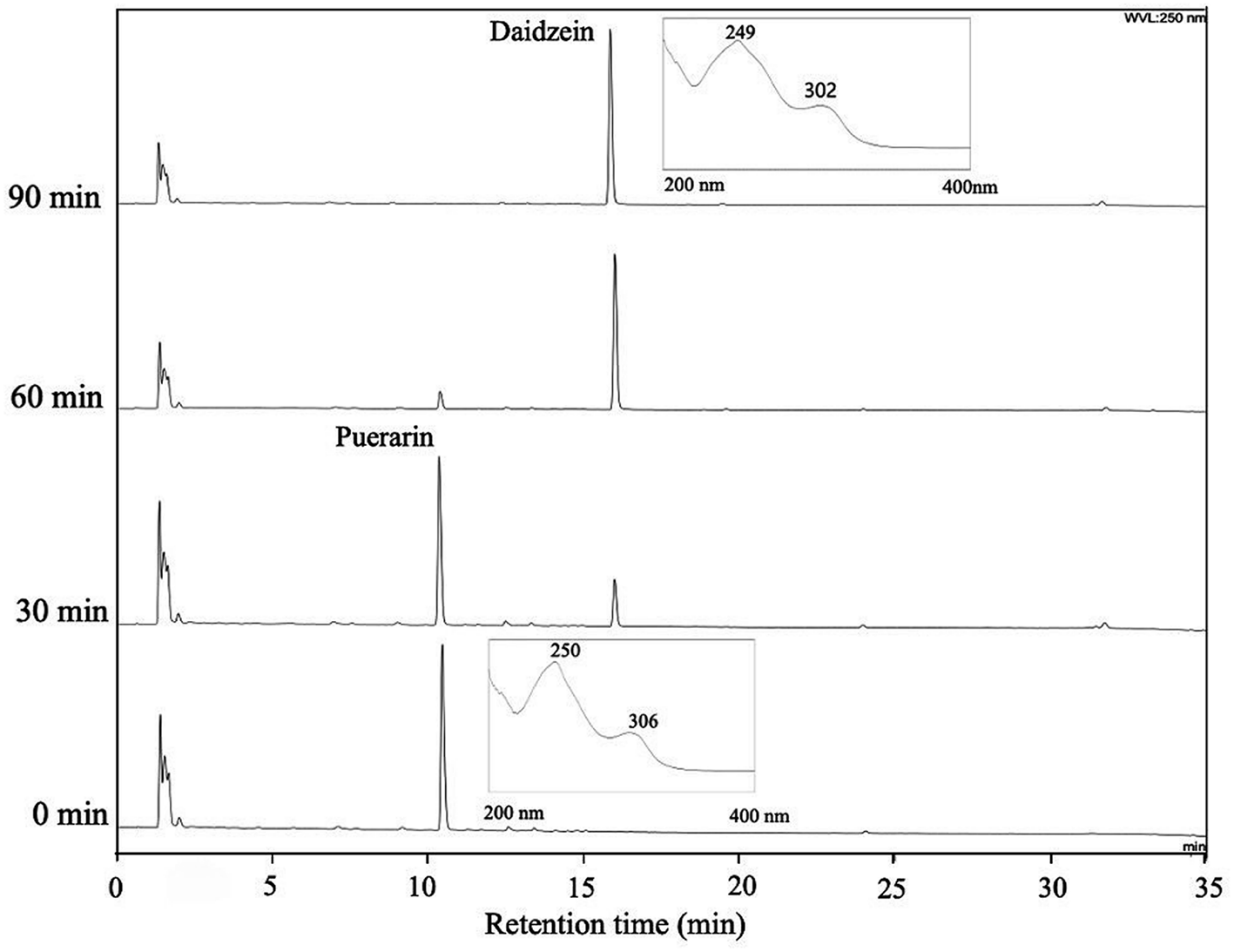
HPLC chromatograms of puerarin biotransformation by *Dorea* sp. MRG-IFC3. Responses were recorded at a wavelength of 250 nm.

**Fig. 4:**
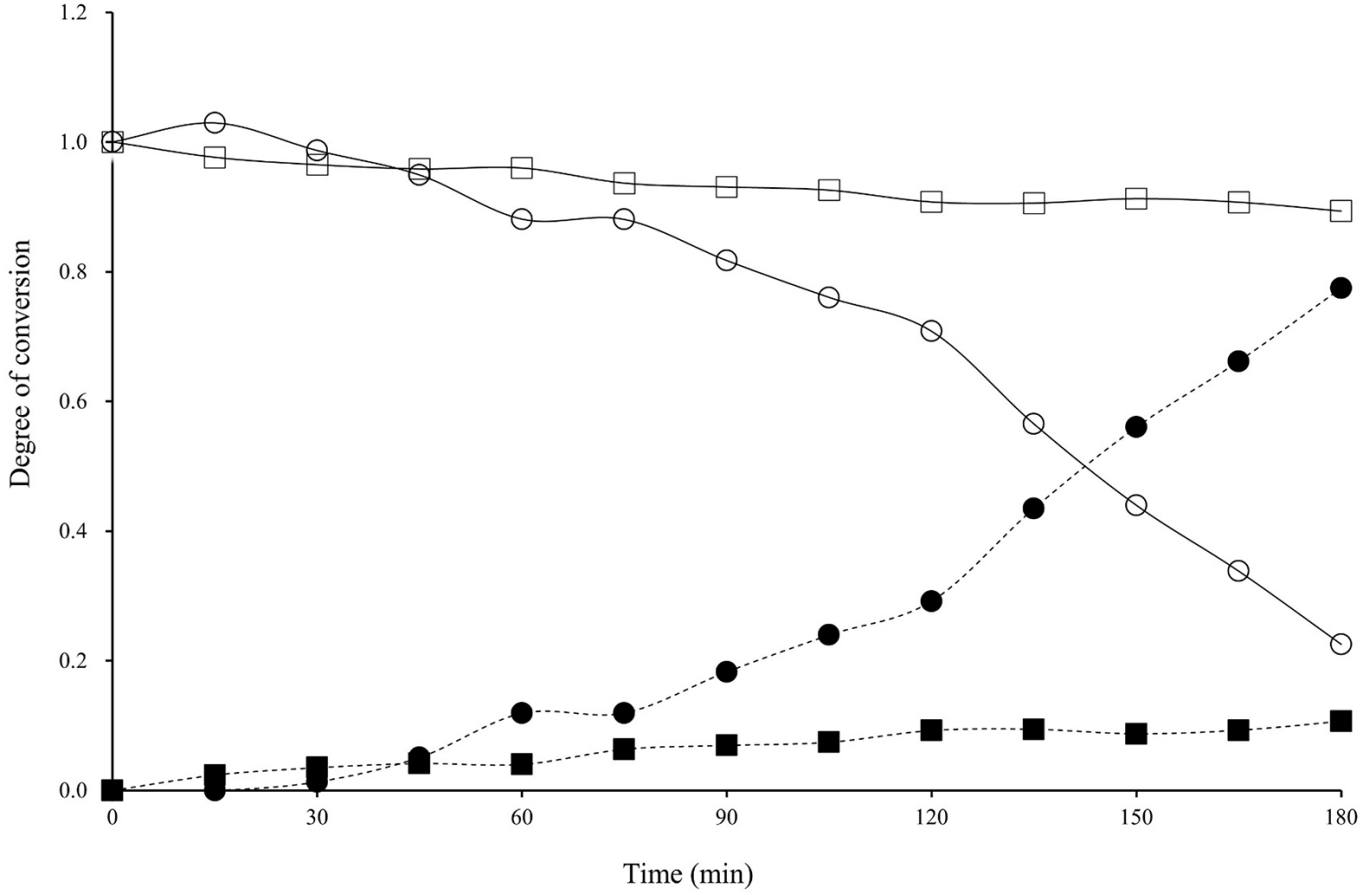
Time-dependent conversion of puerarin by *Dorea* sp. MRG-IFC3. Open circles and squares represent puerarin and daidzin, respectively. Closed circles and squares represent daidzein produced from puerarin and daidzin, respectively.

The rate of puerarin conversion to daidzein was monitored by HPLC analysis (Fig. 3). The peak corresponding to puerarin was observed at a retention time of 10.5 min, and that of daidzein was found at a retention time of 16.0 min, as confirmed by reference compounds. The conversion was slow for the first 30 min, but most puerarin was converted to daidzein in the next 30 min and no substrate was found after 90 min. When time-dependent conversions of puerarin and daidzin were compared (Fig. 4), the biotransformation of puerarin was much faster than that of daidzin. Daidzin was converted to daidzein after 60 min of incubation time, and completely transformed in 180 min. It was suspected that the enzymes responsible for the puerarin metabolism to daidzein were inducible. By comparison with a previous report, the rate of puerarin conversion by MRG-IFC3 was slower than that by MRG-IFC1, but faster than that by MRG-IFC2 [11].

### Biotransformation of Puerarin in D_2_O

**Fig. 5:**
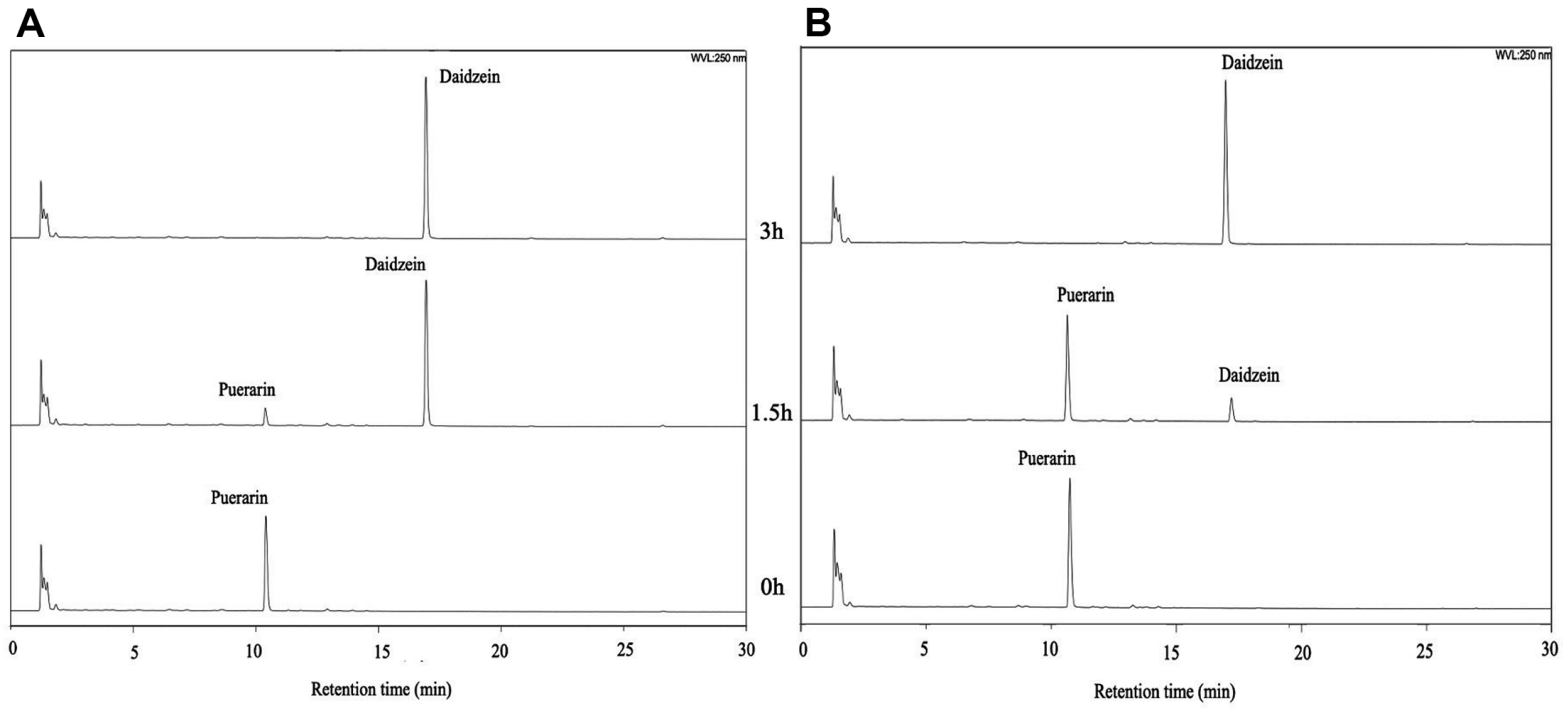
Biotransformation of puerarin by *Dorea* sp. MRG-IFC3 in H_2_O (A) and D_2_O (B) analyzed by HPLC.

GAM media in H_2_O and D_2_O were sterilized by microfiltration for the D_2_O study, since autoclaving would deplete the isotope enrichment. When *Dorea* sp. MRG-IFC3 strain was inoculated and cultured in D_2_O-saturated GAM medium, bacterial growth was a bit slower than conventional culture, but the puerarin biotransformation ability was maintained (Figs. S5 and S6). Puerarin (0.2 mM) was completely changed to daidzein in normal GAM medium in 90 min, while it took 180 min in D_2_O GAM (Fig. 5). The results indicate that the reaction of puerarin with MRG-IFC3 was faster than that in H_2_O under anaerobic conditions at 37°C.

**Fig. 6:**
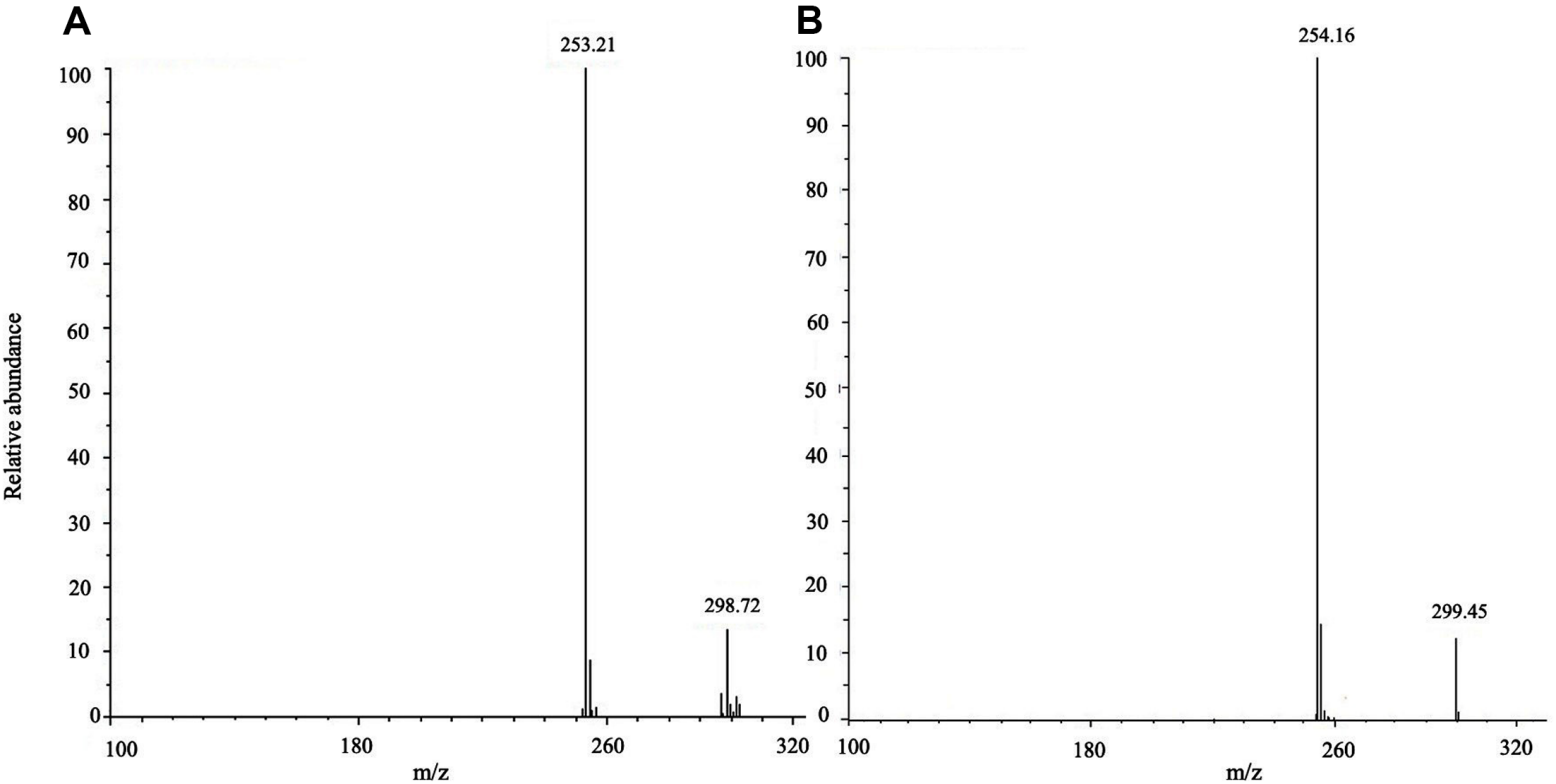
Mass spectra of daidzein metabolites produced in H_2_O GAM (A) and D_2_O GAM (B).

**Fig. 7:**
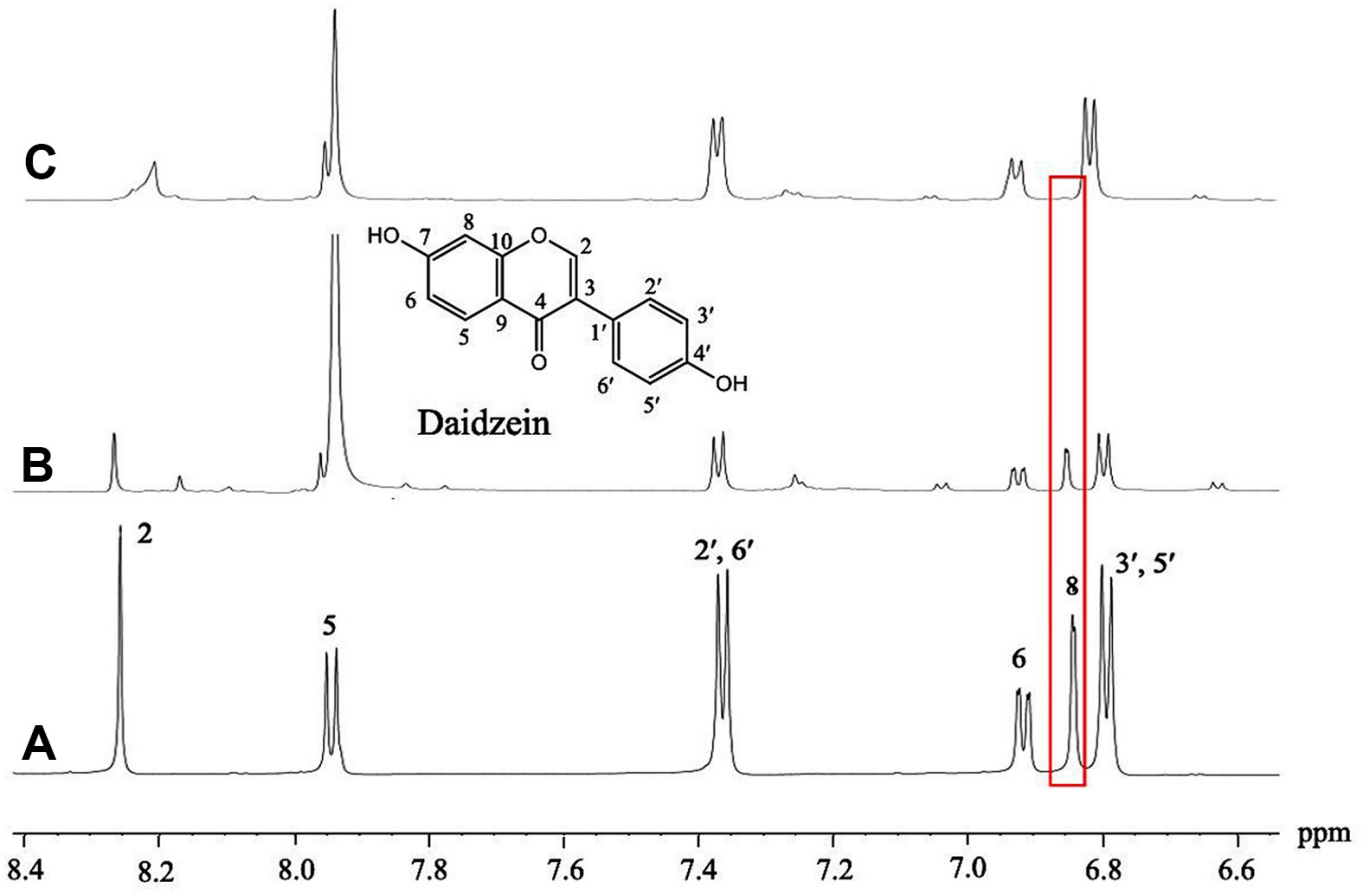
NMR spectra of daidzein were compared. (**A**) Daidzein standard, (**B**) daidzein isolated from the puerarin biotransformation in H_2_O by *Dorea* sp. MRG-IFC3, and (**C**) daidzein isolated from puerarin biotransformation in D_2_O.

The metabolite isolated from the puerarin conversion in D_2_O medium exhibited the same chromatographic behaviors as daidzein on silica gel TLC and HPLC. Subsequently, it was analyzed by HPLC-MS and NMR to check the deuterium enrichment and position of the isotope. The metabolite showed an increase of the molecular ion peak by 1 *m/z* on the MS spectrum, confirming one deuterium incorporation to the daidzein (Fig. 6). Further, the position of deuterium incorporation was clearly identified by ^1^H NMR spectroscopy (Figs. 7, S7, S8, and S9). From the comparison of NMR spectra obtained from standard daidzein (Fig. 7A) and the metabolite isolated from the biotransformation of *Dorea* sp. MRG-IFC3 (Fig. 7B), it was unambiguously confirmed that daidzein was produced from the biotransformation. When the two NMR spectra were compared (Figs. 7B and 7C), complete deuterium exchange was observed at C8-proton, as evidenced by the disappearance of the peak at δ 6.84 ppm.

**Fig. 8:**
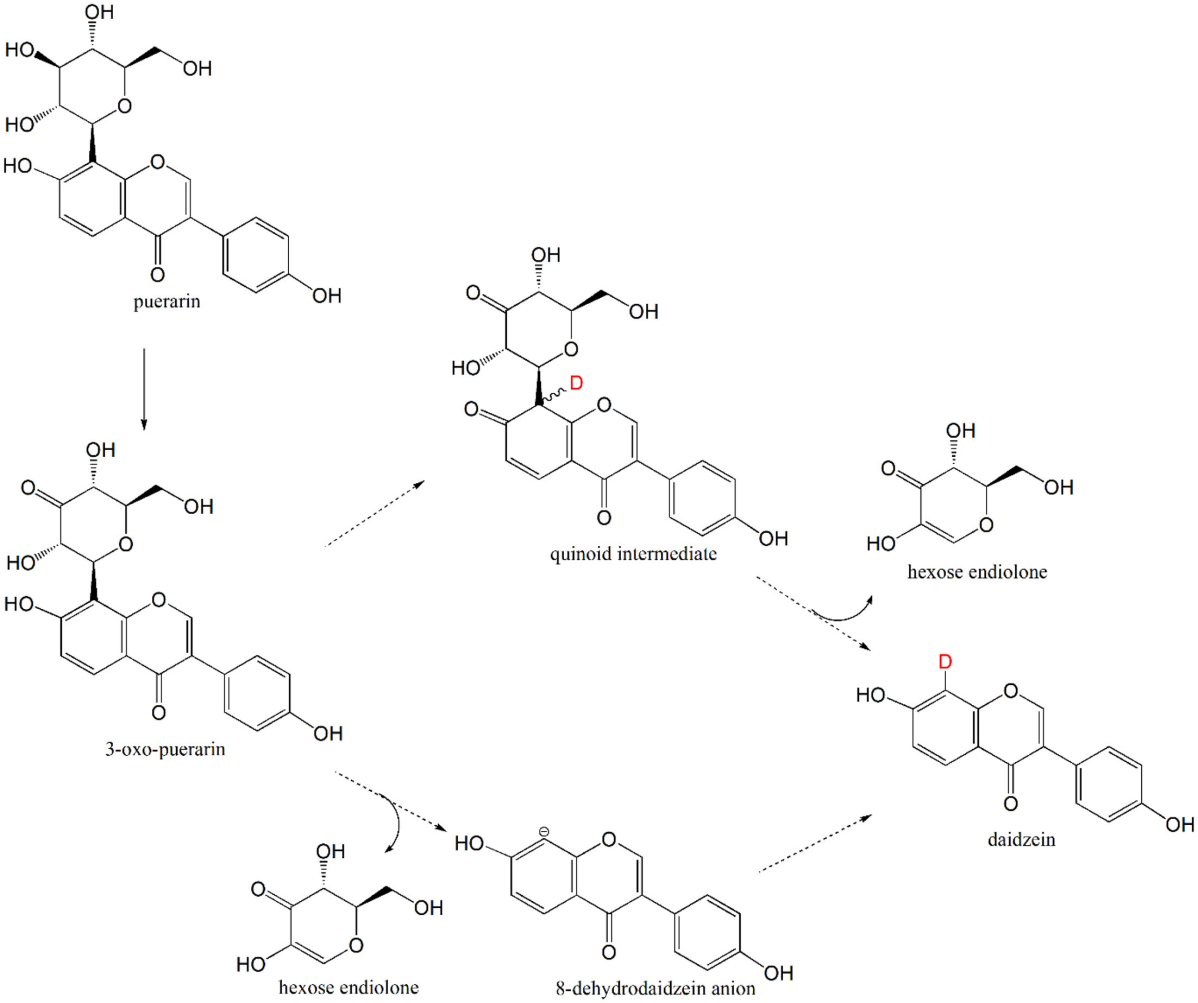
Proposed mechanism of daidzein 8-*d* formation. Puerarin is converted to 3-oxo-puerarin, which can form quinoid intermediates according to the previous proposal [19] or 8-*d*ehydrodaidzein anion through the direct cleavage of C8-C1” glycosidic bond. Both intermediates will lead to the formation of daidzein-8*d* in D_2_O environment.

The result clearly demonstrated that daidzein is directly produced from puerarin biotransformation. Furthermore, identification of daidzein 8-*d* provided mechanistic information regarding the glycosidic C-C bond cleavage of puerarin metabolism. Specifically, the hydrogen atom addition on the C8 position of daidzein is achieved by protonation on the aromatic A-ring of daidzein during the reaction. The protonation step can occur before or after the glycosidic C-C bond cleavage step. In the former scenario, the quinoid intermediate would form [19], whereas in the latter case, 8-*d*ehydrodaidzein anion would form as an intermediate (Fig. 8).

## Discussion

Our recent research has been focused on the discovery of new biochemical conversions carried out by human gut bacteria [2]. The impetus was based on our assumption that the microcosm of the human gut should provide every biochemical pathway for the microbial ecosystem within it. For example, methyl aryl ether cleavage by MRG-PMF1 catalyzed by cobalamin-dependent enzyme [25] produced bioactive demethylated curcuminoids and polymethoxyflavones [23, 26, 27].

In this report, we set out to study new chemical conversions by human gut microbiota. Though various gut bacteria have been reported to metabolize *C*-glycosides by cleaving the glycosidic C-C bond, understanding of the biochemical mechanism for biotechnological application was still incomplete. In this work, a new gut bacterium from a healthy Asian female was screened and identified as *Dorea* sp. The isolated strain MRG-IFC3 could completely convert puerarin to daidzein within a few hours. The conversion rate was slower than *Lactococcus* sp. MRG-IFC-1 but faster than *Enterococcus* sp. MRG-IFC-2 [11]. The phylogenetic tree analysis of MRG-IFC3 showed it is closely related to strain PUE; however, the reactivity of MRG-IFC3 on puerarin was much faster than that of PUE strain based on the reported data [14]. Interestingly, *Enterococcus faecalis* W12-1 could transform flavone *C*-glycosides orientin, vitexin, and isovitexin to their corresponding aglycones, but did not cleave isoflavone *C*-glycoside puerarin to daidzein [13]. In contrast, *Dorea* sp. MRG-IFC3 cleaved puerarin to daidzein but not vitexin, similar to MRG-IFC1 and MRG-IFC2. *Lachnospiraceae* strain CG19-1 was also reported to show a broad substrate activity on both flavone and isoflavone *C*-glycosides, including puerarin, homoorientin, and vitexin [12]. Although it is not clear whether each bacterium expresses different enzymes for C-glycosidic bond cleavage, it suggested that *C*-glycoside bioconversion is universal in nature.

The results obtained from the conversion of puerarin in D_2_O-substituted medium confirmed complete deuterium labeling at the C8 position of daidzein. Accordingly, it demonstrates that the identified metabolite daidzein is produced directly from the substrate puerarin. More importantly, the identification of daidzein 8-*d* provided structural information on the reaction intermediates formed during the C-C bond cleavage of puerarin (Fig. 8). Deprotonation of 7-OH group of 3-oxo-puerarin could lead to the formation of quinoids, whereas the direct cleavage of the glycosidic C-C bond would also lead to the formation of the daidzein 8C anion (8-*d*ehydrodaidzein anion) species. To investigate which mechanism is responsible for the production of daidzein 8-*d*, biotransformation study should be performed with the substrate derivatives, such as 7-fluoropuerarin.

Gut metabolism of *C*-glycosides is of great significance due to the metabolism of bioactive *C*-glycosides in vivo and the chemically unprecedented C-C bond cleavage reaction between the anomeric carbon and the aryl group of aglycone. The newly isolated *Dorea* sp. MRG-IFC3 should enable us to investigate *C*-glycoside gut metabolism, especially the unique C-C bond cleavage reaction, at the molecular level. In this study, two possible reaction intermediates of the glycosyl C-C bond cleavage reaction, the quinoids and 8-*d*ehydrodaidzein anion, were proposed from the puerarin biotransformation in D_2_O medium.

## Supplemental Materials

Supplementary data for this paper are available on-line only at http://jmb.or.kr.



## Figures and Tables

**Table 1:** Reactivity of *Dorea* sp. MRG-IFC3.

Substrate	Metabolite	Reactivity
Puerarin	Daidzein	~ 42% metabolite formation within 60 min, completely metabolized within 90 min.
Aloin	NC	-
Bergenin	NC	-
Vitexin	NC	-
Apigetrin	Apigenin	~ 43% substrate conversion within 6 h, substrate was completely metabolized after 24 h reaction time.
Daidzin	Daidzein	~ 69% metabolite formation within 48 h, daidzin almost metabolized all within 96 h.
Glycitin	Glycitein	~ 56% within 24 h and ~79% within 48 h metabolite formation.
Genistin	Genistein	~ 50% within 3 h and ~79% within 6 h metabolite formation, completely metabolized within 48 h.
Icariin	Icarisides II	~65% within 6 h and ~ 87% within 24 h metabolite formation, completely metabolized within 96 h.
Icarsides II	NC	-
Ononin	Formononetin	~59% within 3 h and ~ 88% within 6 h metabolite formation, completely metabolized within 24 h.
Sissotrin	Biochanin A	completely metabolized within 3 h.

NC: No conversion
