# Supplementary material for: C-Glycoside-Metabolizing Human Gut Bacterium, Dorea sp. MRG-IFC3
Source: J Microbiol Biotechnol. 2023 Sep 21;33(12):1606–14. doi: 10.4014/jmb.2308.08021 (PMC10772555; doi:10.4014/jmb.2308.08021)
Supplement: Supplementary file 1 [file jmb-33-12-1606-supple.pdf]

J. Microbiol. Biotechnol.
https://doi.org/10.4014/jmb.2308.08021

Supplementary Figures and Table

C-Glycoside metabolizing human gut bacterium, *Dorea* sp. MRG-IFC3

Huynh Thi Ngoc Mi, Santipap Chaiyasarn, Heji Kim and Jaehong Han*

Metalloenzyme Research Group and Department of Plant Science and Technology, Chung-Ang University, 4726 Seodong-daero, Anseong 17546, Republic of Korea

Corresponding author

E-mail: jaehongh@cau.ac.kr

Morphological analysis

Isolated bacteria were streaked on the GAM agar plate and incubated for 24 hours under anaerobic conditions. The colony was taken for a Gram staining test using Gram staining Kit (Sigma-Aldrich, Buchs-Switzerland). A single colony was picked and spread in a thin film over a glass slide with a drop of distilled water, air-dried, and then passed through the flame to fix onto the glass slide. Primary staining was conducted by dropping crystal violet onto the heat-fixed smear for 1 minute. After washing with distilled water, iodine was added for 1 minute, wash then with distilled water. The smear was applied 95% ethanol to decolorize for 10-20 seconds. Secondary staining was performed with safranin for 1-2 minutes. Drain and wash with distilled water. The cells were observed by microscopy (100x objective).

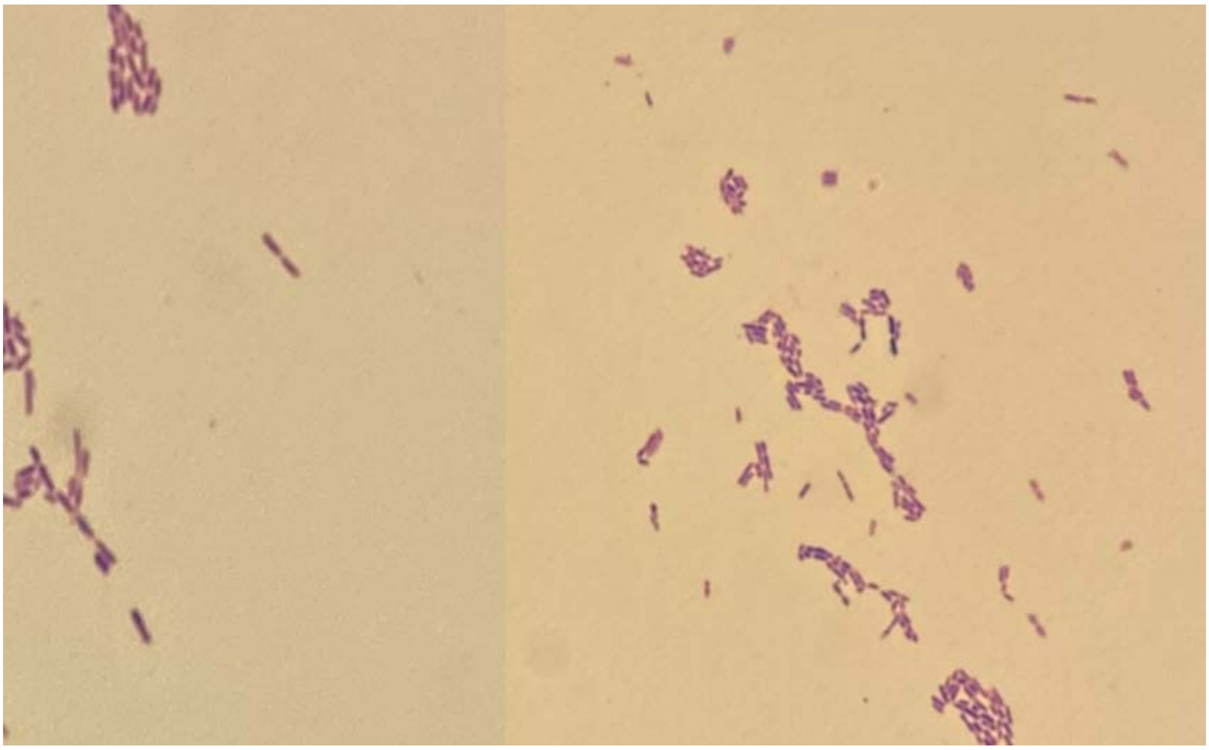

Figure S1. Gram staining image of *Dorea* sp. MRG-IFC3.

Air-sensitivity of bacterial growth

Inoculate 50 μL of the prepared bacteria in 5 mL GAM broth inside an anaerobic chamber. Prepare two samples to grow inside and outside the chamber, measure the OD of bacteria at the 2 conditions for comparing.

Table S1. The OD 600 nm of *Dorea* sp. MRG-IFC3 grown in air conditions and anaerobic conditions.

Incubation time	Air conditions at room temperature	Anaerobic conditions at 37°C
**0h**	0.0187	0.0595
**6h**	0.0213	0.6701
**12h**	0.0019	2.0942
**24h**	0.0750	2.2365

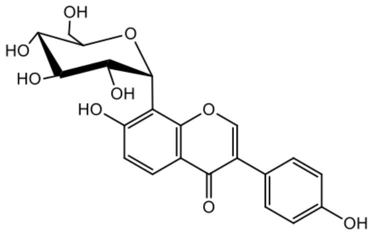

Puerarin

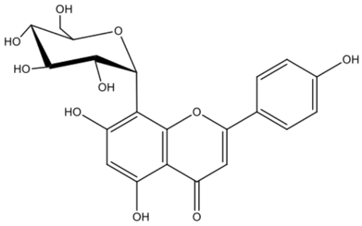

Vitexin

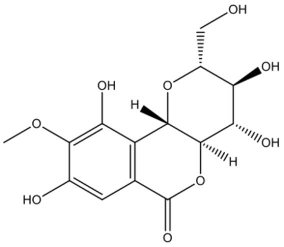

Bergenin

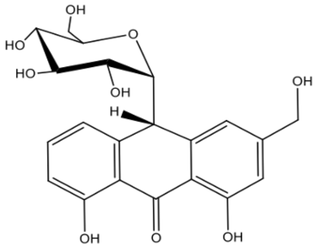

Aloin

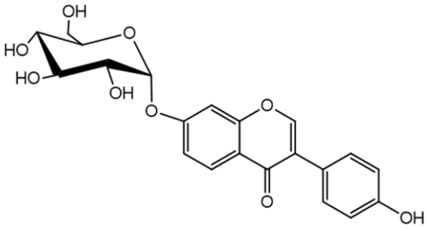

Daidzin

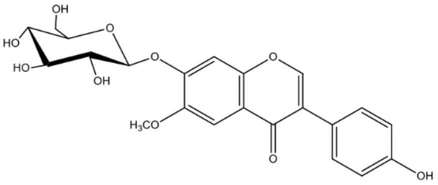

Glycitin

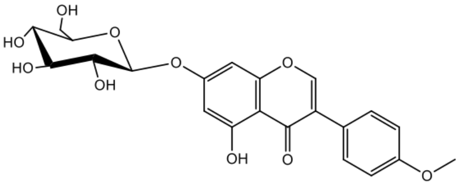

Sissotrin

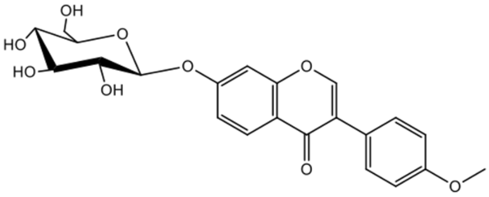

Ononin

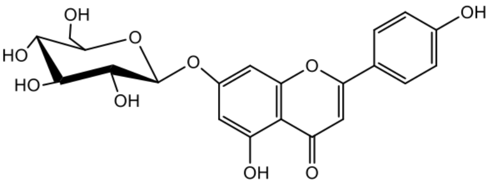

Apigetrin

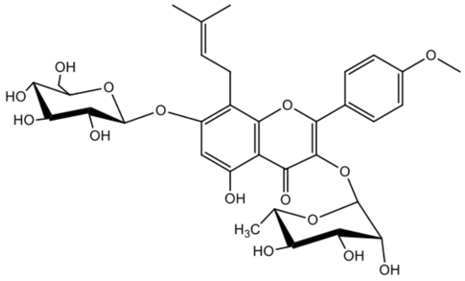

Icariin

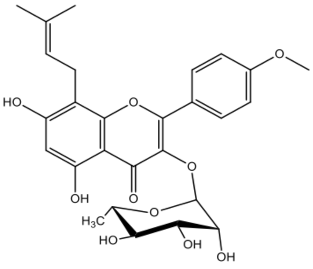

Icariside II

Figure S2. Molecular structure of substrate reacted with MRG-IFC3 strain.

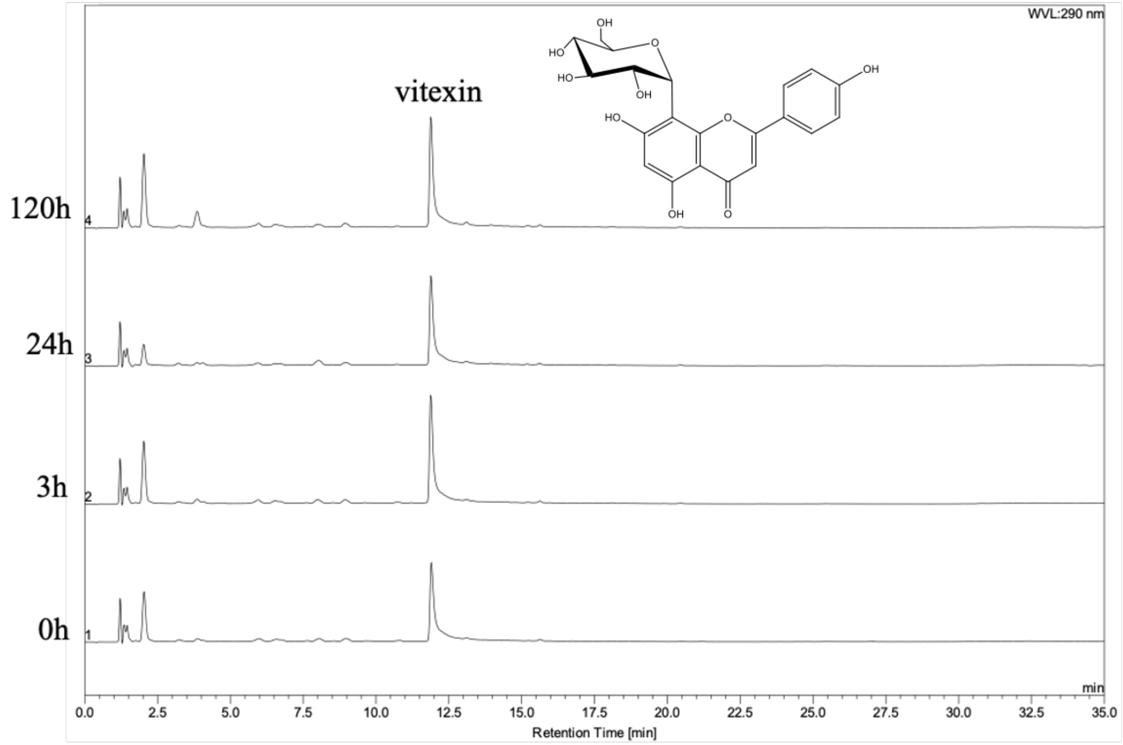

Figure S3. Chromatograms profile of biotransformation of vitexin by MRG-IFC3. Responses were recorded at wavelength of 290nm.

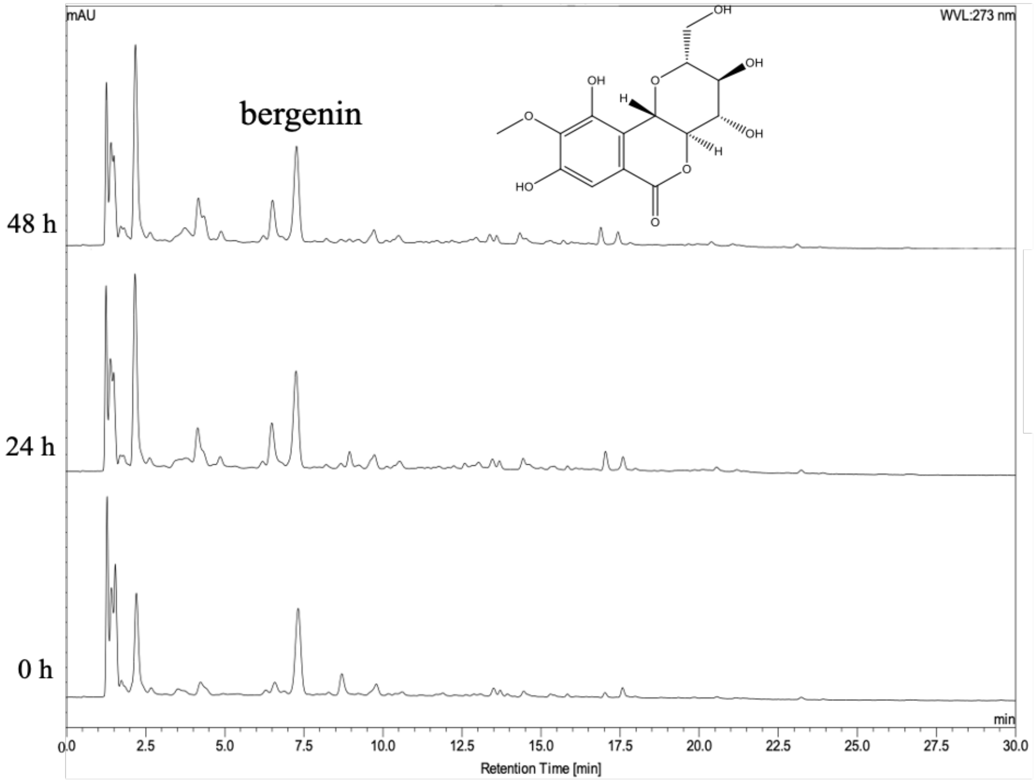

Figure S4. Chromatograms profile of biotransformation of bergenin. Responses were recorded at a wavelength of 273 nm.

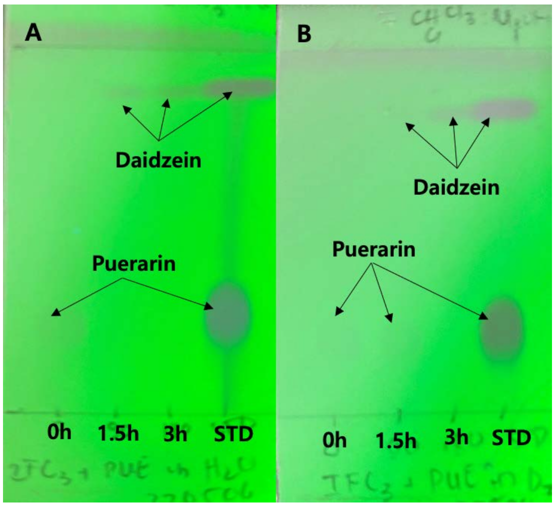

Figure S5. Thin-layer chromatography (TLC) profile of biotransformation of Puerarin by MRG-IFC-3 under short-wavelength UV light (254 nm). **A**: reacted in H_2O; **B**: reacted in D_2O. The solvent system used in this assay is $CHCl_3$: MeOH (4:1).

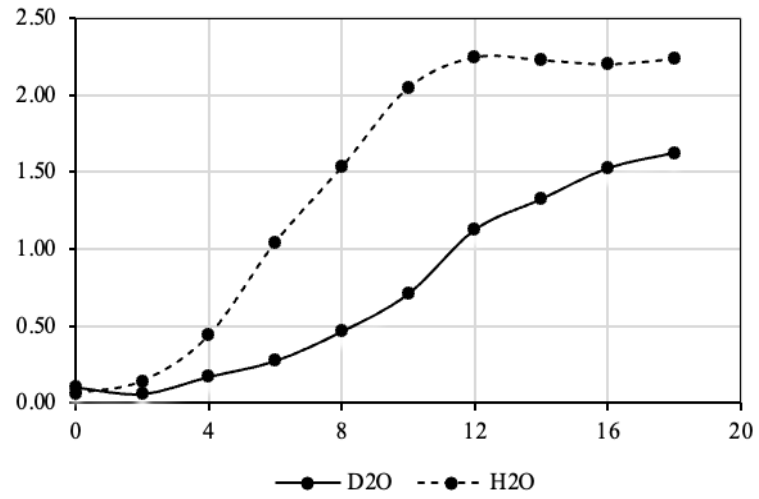

Figure S6. Growth curve of *Dorea* sp. MRG- IFC3 in D_2O and H_2O. Cell growth was measured by monitoring OD_{600} in GAM medium at 37 °C under anaerobic conditions.

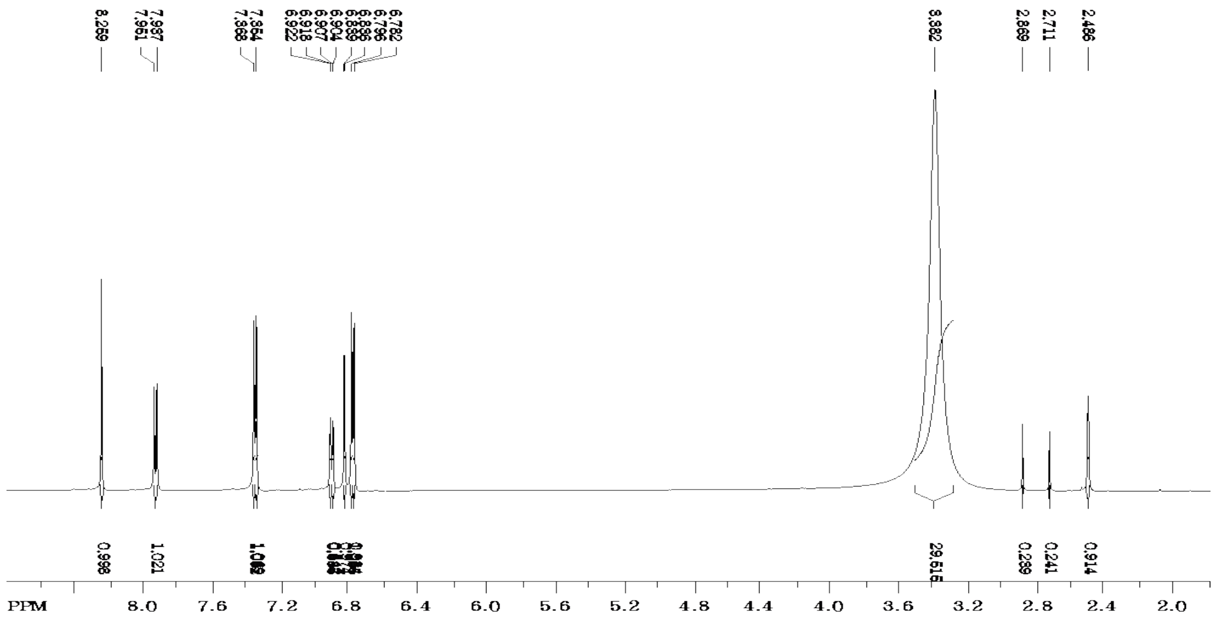

Figure S7. ^{1}H-NMR spectrum of daidzein standard.

DMSO, 600MHz: δ 8.3 (s, 1H, C2), 7.94 (d, J=8.4Hz, 1H, -CH=, C5); 7.36 (d, J=8.4Hz, 1H,-CH=, C2′, C6′); 6.91 (dd, J=1.8Hz, 8.4Hz, C6); 6.84 (d, 1H, C8), 6.80 (d, J=8.4Hz, C3′, C5′).

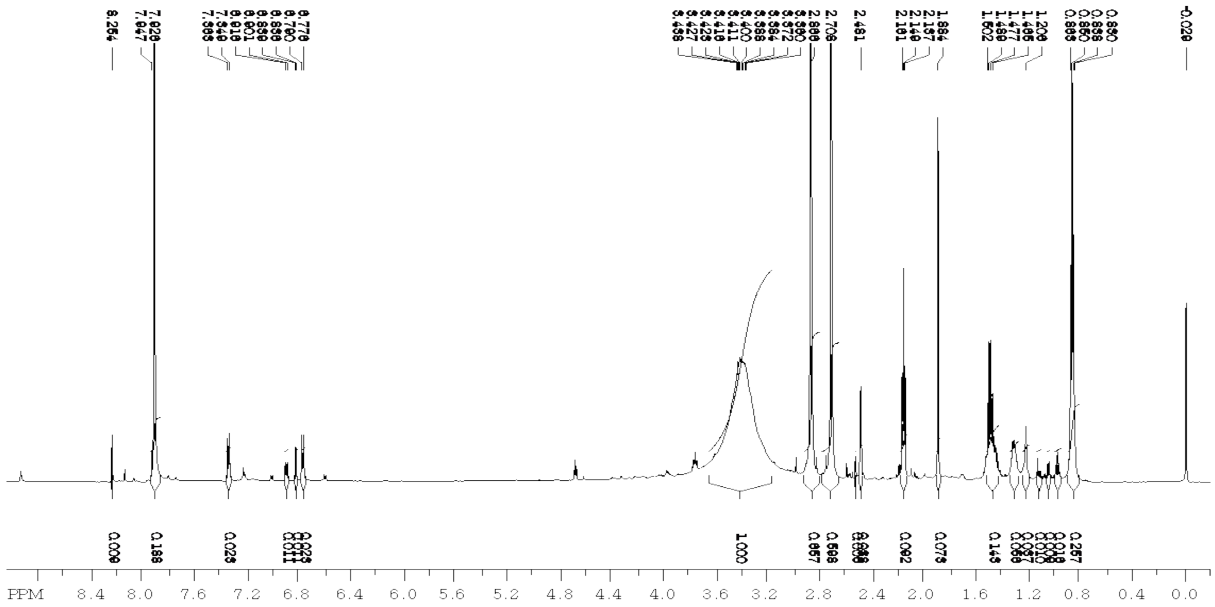

Figure S8. ^{1}H-NMR spectrum of daidzein produced in H_2O.

DMSO, 600MHz: δ 8.3 (s, 1H, C2), 7.94 (d, J=8.4Hz, 1H, -CH=, C5); 7.36 (d, J=8.4Hz, 1H,-CH=, C2′, C6′); 6.91 (dd, J=1.8Hz, 8.4Hz, C6); 6.84 (d, 1H, C8), 6.80 (d, J=8.4Hz, C3′, C5′).

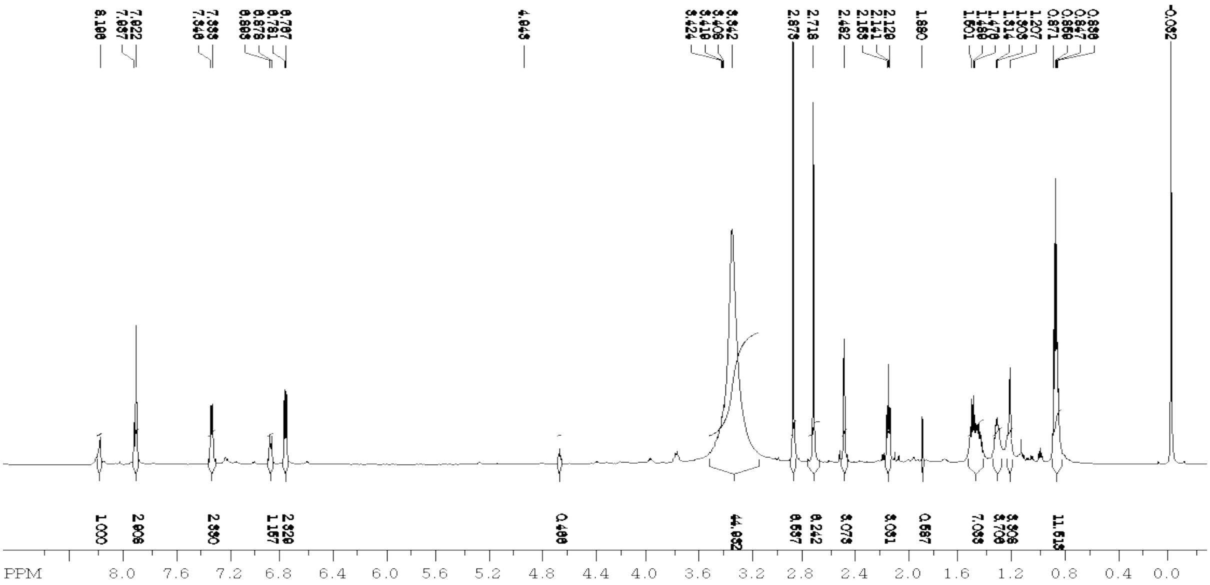

Figure S9. ^{1}H-NMR spectrum of daidzein produced in D_2O.

DMSO, 600MHz: δ 8.3 (s, 1H, C2), 7.94 (d, J=8.4Hz, 1H, -CH=, C5); 7.36 (d, J=8.4Hz, 1H,-CH=, C2′, C6′); 6.90 (d, J=9Hz, C6);6.80 (d, J=8.4Hz, C3′, C5′).
